# Predictive factors for skeletal complications in hormone-refractory prostate cancer patients with metastatic bone disease

**DOI:** 10.1038/sj.bjc.6602767

**Published:** 2005-09-13

**Authors:** A Berruti, M Tucci, A Mosca, R Tarabuzzi, G Gorzegno, C Terrone, F Vana, G Lamanna, M Tampellini, F Porpiglia, A Angeli, R M Scarpa, L Dogliotti

**Affiliations:** 1 Dipartimento di Scienze Cliniche e Biologiche Università degli Studi di Torino, Prostate Cancer Unit, Oncologia Medica, Urologia, Medicina Interna, Azienda Ospedaliera San Luigi, Orbassano, Italy

**Keywords:** prostate cancer, bone metastases, adverse skeletal events

## Abstract

Factors predictive of skeletal-related events (SREs) in bone metastatic prostate cancer patients with hormone-refractory disease were investigated. We evaluated the frequency of SREs in 200 hormone-refractory patients consecutively observed at our Institution and followed until death or the last follow-up. Baseline parameters were evaluated in univariate and multivariate analysis as potential predictive factors of SREs. Skeletal-related events were observed in 86 patients (43.0%), 10 of which (5.0%) occurred before the onset of hormone-refractory disease. In univariate analysis, patient performance status (*P*=0.002), disease extent (DE) in bone (*P*=0.0001), bone pain (*P*=0.0001), serum alkaline phosphatase (*P*=0.0001) and urinary N-telopeptide of type one collagen (*P*=0.0001) directly correlated with a greater risk to develop SREs, whereas Gleason score at diagnosis, serum PSA, Hb, serum albumin, serum calcium, types of bone lesions and duration of androgen deprivation therapy did not. Both DE in bone (hazard ratio (HR): 1.16, 95% confidence interval (CI): 1.07–1.25, *P*=0.000) and pain score (HR: 1.13, 95% CI: 1.06–1.20, *P*=0.000) were independent variables predicting for the onset of SREs in multivariate analysis. In patients with heavy tumour load in bone and great bone pain, the percentage of SREs was almost twice as high as (26 *vs* 52%, *P*<0.02) and occurred significantly earlier (*P*=0.000) than SREs in patients with limited DE in bone and low pain. Bone pain and DE in bone independently predict the occurrence of SREs in bone metastatic prostate cancer patients with hormone-refractory disease. These findings could help physicians in tailoring the skeletal follow-up most appropriate to individual patients and may prove useful for stratifying patients enrolled in bisphosphonate clinical trials.

Prostate cancer is the most commonly diagnosed malignancy among elderly men and the second most common cause of cancer-related death in men in Italy and other European countries (De Angelis *et al*, 1997). In patients with prostate cancer, bone is by far the most common site of distant metastases. More than 80% of men with advanced disease will, in fact, eventually develop skeletal involvement. Bone metastases are devastating events in the natural history of the disease since they are often painful and cause skeletal complications such as pathologic fracture, vertebral collapse and spinal cord compression (Berruti *et al*, 2000; Bayley *et al*, 2001; Mundy, 2002). Androgen suppression by either orchiectomy or administration of luteinising hormone-releasing hormone analogs (LHRH-A) is the mainstay of treatment for advanced prostate cancer patients. This therapy frequently produces tumour shrinkage and improvement in symptoms, but it is not curative since almost all patients will eventually develop hormone-refractory disease. Once the disease becomes refractory to hormonal manipulation, the prognosis is dismal and the overall survival is about 13–16 months (Smaletz *et al*, 2002; Halabi *et al*, 2003).

We have previously observed that skeletal-related events (SREs) in prostate cancer patients are relatively rare as long as the disease is responsive to hormones, but they occur much more often when the tumour becomes hormone refractory (Berruti *et al*, 2000). Zoledronic acid, a potent bisphosphonate, significantly reduces the onset of skeletal complications in this patient population (Saad *et al*, 2002). Recent reports point out, however, that administration of this drug is not cost-effective (Reed *et al*, 2004). Other available treatment options include intensive supportive care, radiotherapy, second-line hormonal manipulations, cytotoxic chemotherapy and investigational agents (Goodin *et al*, 2002). In two recent randomised independent clinical trials, chemotherapy reportedly improved response rate and survival (Petrylak *et al*, 2004; Tannock *et al*, 2004). However, because the survival benefit is modest, treatment options for bone metastatic patients with hormone-refractory disease are mainly palliative.

Since skeletal complications are the major causes of diminished quality of life in this patient population (Groot *et al*, 2003), the identification of baseline factors predictive for these adverse events may be a valuable aid in tailoring to each patient the most appropriate preventive measures and in potentially avoiding unnecessary and costly treatments.

In advanced prostate cancer, the basic factors contributing to skeletal-related morbidity may be broken down loosely into those related directly to the metastases and those of iatrogenic nature. Androgen deprivation therapy causes osteoporosis and adverse skeletal events independently of disease response and in the absence of skeletal involvement (Daniell *et al*, 2000; Smith, 2003). The frequency of adverse skeletal events induced by androgen deprivation therapy depends mainly on the duration of therapy. Long-lasting androgen deprivation therapy makes the skeleton more fragile and may contribute to skeletal morbidity even in the hormone-refractory phase of the disease.

In this study, the frequency of SREs was evaluated in a series of hormone-refractory prostate cancer patients with metastatic bone disease prospectively treated and followed at the Prostate Cancer Unit of the Azienda Ospedaliera San Luigi di Orbassano, Italy. In a previous publication on the first 112 cases (Berruti *et al*, 2000), we identified several predictive factors, including serum alkaline phosphatase (ALP), urinary deoxy-pyridinoline and disease extent (DE) in bone. Here, we tested by univariate and multivariate analyses a number of additional predictive factors for SREs, including validated prognostic parameters, urinary *N*-telopeptide of type one collagen (NTx) and duration of androgen deprivation therapy.

## PATIENTS AND METHODS

### Patients

Eligible patients were required to have histologically proven adenocarcinoma of the prostate with metastatic bone disease that progressed to LHRH-A administration with or without antiandrogens. Disease progression was defined as a rise in prostate-specific antigen (PSA) levels (⩾50% over the nadir obtained during androgen deprivation) on two consecutive measurements performed at least 2 weeks apart or a new lesion on bone scan and/or an increase in the size of a measurable lesion on a computed tomographic (CT) scan of the abdomen/pelvis or chest. The time from diagnosis of hormone refractoriness to enrolment in the study should have been not greater than 2 months.

Patients previously treated with LHRH-A alone underwent total androgen ablation (obtained by adding an antiandrogen: flutamide or bicalutamide). They entered the study if they showed further evidence of disease progression after at least 4 weeks of combined therapy. Patients previously treated with total androgen ablation were required to undergo antiandrogen withdrawal. They were required to be off all antiandrogens for at least 4 weeks, with further evidence of disease progression after cessation of antiandrogen treatment. Serum testosterone was routinely measured in all patients showing PSA progression under LHRH-A since 1997. From this date onwards, testosterone inhibition (defined as serum levels <50 ng dl^−1^) was added to the criteria for hormone refractoriness. Further inclusion criteria comprised an Eastern Cooperative Oncology Group (ECOG) performance status (PS) of 0–3 and normal renal and hepatic function. Patients were excluded from the study if they had severe uncontrolled comorbidity, second malignancies, pretreatment with bisphosphonates, radiotherapy or radionuclide therapy for palliation of bone pain, and second-line antineoplastic treatment. At study entry, all patients underwent physical examination, including bone pain assessment and neurological examination, routine blood chemistry studies, serum PSA, serum ALP, bone scan followed by radiological confirmation of hot spots, chest X-ray and whole abdomen CT. Urinary NTx was measured in the last 103 patients. When scintigraphy was positive and X-ray films were negative for bone lesions, a CT study was performed. The whole skeleton was arbitrarily divided into the following areas: skull, cervical, dorsal, lumbar spine+sacrum, right leg, left leg, right arm, left arm, right ribs, left ribs, sternum, right scapula and clavicula, left scapula and clavicula, right pelvis and left pelvis. Disease extent in bone was calculated as the sum of involved areas. In order to limit the distribution of this variable, patients with ⩾9 segments were scored as 9. Baseline bone pain was evaluated using a validated pain questionnaire, as previously reported (Berruti *et al*, 2002). Questionnaire items included performance status, analgesic consumption and mobility, as measured on a pain score of 0–19. All patients subsequently received second-line treatment consisting of endocrine therapy, chemotherapy, radiotherapy and radionuclide therapy, as indicated in association with the best palliative care. Physical and bone pain reassessment, routine blood chemistry studies, bone markers and serum PSA were repeated at least every 3 months. On detection of increased PSA, worsening bone pain and/or impaired mobility, patients underwent bone scanning followed by radiological confirmation of hot spots. When pathological or impending fracture was diagnosed, patients were referred to an orthopaedic surgeon. When deformity of 1 or more vertebral bodies was detected or clinical evidence of spinal cord compression was suspected, whole spine magnetic resonance imaging (MRI) was performed to direct decision-making whether to perform surgery or radiotherapy and/or prescribe spinal orthosis. The study was approved by the Institutional Review Board of our hospital. All patients were required to give written informed consent before registration.

### Study end points

Skeletal-related events were defined as vertebral body collapse requiring spinal orthosis, spinal cord compression, vertebral and nonvertebral pathological fractures, symptomatic hypercalcemia, symptomatic hypocalcemia. Skeletal-related events also included surgery and/or radiotherapy delivered to bone to stabilise or prevent pathologic fractures or areas of spinal cord compression. Neither radiation therapy nor radionuclide therapy for pain relief nor change in antineoplastic therapy to treat bone pain were considered SREs. Survival duration was defined as the time between the diagnosis of hormone-refractory disease and death. The date of hormone refractoriness was defined as the date of the first rise in PSA or, in the rare cases of no PSA elevation, the date of diagnosis of disease progression in skeletal or extraskeletal sites. Patients were censored if they were known to be alive or were lost to follow-up. The time to the onset of skeletal complications was calculated as the time between the diagnosis of hormone-refractory disease and the occurrence of the first SRE or death, whichever event occurred first. Patients were censored if they were alive and free of SREs. If the first SRE occurred before the onset of hormone refractoriness, it was not computed in the analysis.

### Biochemical measurements

Early morning spot urine specimens were obtained to measure creatinine and *N*-telopeptide; blood samples were drawn to assess calcium, albumin (ALB), PSA, total ALP, lactate dehydrogenasis (LDH) and haemoglobin (Hb). *N*-telopeptide of type one collagen was measured using a commercial kit (Osteomark, Ortho-Clinical Diagnostics, Rochester, NY, USA), blood biochemical parameters were measured using automated procedures (Architect, Abbott).

### Statistical analysis

Differences in proportion were determined using the *χ*^2^ test. Correlations between continuous variables were performed using the nonparametric Spearman test. Survival curves were estimated using the Kaplan–Meier method. Unadjusted differences in these estimates were tested using the log-rank test. A univariate Cox proportional hazards model was used to identify all statistically significant predictors of SREs and death. A multivariate Cox proportional hazards model was used to identify independent variables predictive of the onset of SREs. Only those variables found to be significant on univariate analysis were included in a multivariate proportional hazards model. Among these, serum ALP and urinary NTx had right skewed distributions and were modelled using log transformation. Two analyses were performed, one on overall cases without including NTx and another on the 103 patients having NTx measured. The latter analysis should be considered as explorative. All reported *P*-values were two-sided; *P*-values <0.05 were considered statistically significant. Statistical computation was performed using the SPSS for Windows software package.

## RESULTS

### Patients

From July 1990 to January 2003, 200 consecutive patients with newly diagnosed hormone-refractory prostate cancer meeting the eligibility criteria were enrolled in the present study. Patient characteristics are depicted in Table 1. Urinary NTx was available for the last 103 patients, while in the first patients bone resorption was measured by urinary deoxypyridinoline. Treatment for hormone-refractory disease was chemotherapy in 74 patients (estramustine alone, etoposide+estramustine, epirubicin, docetaxel) and steroids plus supportive care in the remaining 126 cases. Previous LHRH-A administration was not interrupted in any cases. Bone pain was present in 177 patients (88.5%); 72 (36.0%) necessitated radiotherapy and or radionuclide therapy to control pain symptoms; 32 (16.0%) also received single-dose pamidronate, as previously reported (Berruti *et al*, 2002). As expected, a significant correlation was found between DE in bone and either ALP serum levels (Spearman R 0.51, *P*<0.01) or NTx urinary values (Spearman R 0.40, *P*<0.01).

A total of 86 patients (43.0%) experienced skeletal complications, including vertebral collapse in 41 (20.5%), fractures in 25 (12.5%) and spinal cord compression in 20 (10.0%). Skeletal fractures involved the vertebrae (seven patients), the ribs (six patients), the femur (six patients), the scapula (one patient), the pelvis (three patients), the humerus (one patient), the vertebrae and ribs (one patient). Two patients developed symptomatic hypercalcemia and one patient symptomatic hypocalcemia. Both metabolic adverse events occurred concomitantly with other SREs and were not computed separately.

All patients who experienced SREs received radiation therapy; 13 required surgery to bone. Additional SREs occurring in patients who had already experienced an adverse skeletal event were not considered in the analysis. In 10 (5.0%), the first adverse SRE preceded the onset of hormone refractory disease; in 76 (38.0%) it occurred afterwards. In those cases where SREs occurred before hormone refractoriness, the median duration of androgen deprivation therapy was 59.9 months (range 19.3–228.0) and 24.9 months in those where it did not (range 3.0–212.0, *P*=0.004). In the patients who experienced SREs in the hormone-refractory phase of the disease, the median time to the onset of adverse events was 7 months (range 1–39). At the last follow-up on 15 August 2004, 180 patients (90.0%) had died (median survival 14.5 months); 102 of which (56.7%) without experiencing SREs. Out of 20 patients, 12 still alive were SRE-free, five of which were alive more than 4 years since the date of the onset of hormone-refractory disease.

### Skeletal-related events according to baseline characteristics

The proportion of patients with at least one SRE according to baseline parameters and the univariate relative risks for the onset of SREs and death is shown in Table 2. Skeletal-related events occurring before the onset of hormone-refractory disease were not considered in the analysis. Disease extent in bone, bone pain, serum ALP and urinary NTx directly correlated with a higher risk of developing adverse skeletal events over time (either in terms of proportion of SREs or in terms of relative risk). Analysis of Gleason score, serum PSA, Hb, serum albumin, serum calcium, types of bone lesions and duration of androgen deprivation therapy did not show any significant relationship between these parameters and the occurrence of SREs. Performance status failed to be significantly correlated with the frequency of SREs, while it was significantly related with a higher risk of experiencing an SRE over time.

Those variables attaining statistical significance in the univariate analysis were included in the multivariate analysis. In the first multivariate analysis of all cases, both DE in bone and bone pain were independent variables predicting for SREs, while ALP and patient performance status did not enter the model (Table 3). In the multivariate analysis on patients with NTx assessed alone, DE in bone and bone pain maintained an independent predictive role, while NTx failed to enter the model (HR: 1.13, 95% CI: 0.71–1.77).

**Figure 1:**
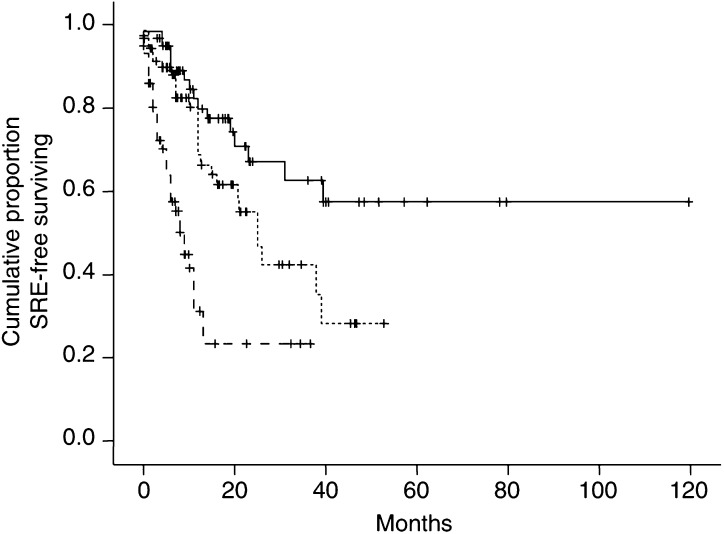
Time to the onset of SRE in patients with low pain and limited disease extent in bone (Pain−/DE−) () *vs* patients with great bone pain and heavy tumour load in bone (Pain+/DE+) (), *vs* patients with either of these two predictors ().

Disease extent in bone and bone pain (both considered dichotomous variables as outlined in Table 1) were combined to generate three patient subgroups: patients with DE in ⩽6 sites and a pain score of <5 (DE−/Pain−), patients with DE in >6 sites and a pain score of ⩾5 (Pain+/DE+) and patients with either of these predictors (DE−/Pain+ or DE+/Pain−). The distribution of SREs in patients with Pain+/DE+ was double that of patients with Pain−/DE− (26.2 *vs* 52.5%, respectively, *P*=0.016), while the SRE frequency in patients with either of these two predictors was intermediate (36.0%). Analysis of the time to onset of first SRE also showed a time-dependent pattern (Figure 1). Patients with heavy tumour load in bone and great bone pain (Pain+/DE+) developed SREs significantly earlier than those with limited DE in bone and low pain (Pain−/DE−) (RR: 1.63, 95% CI: 1.32–2.00). Again, patients with either predictor showed an intermediate pattern.

All validated prognostic parameters such as Gleason score, serum albumin, serum ALP, LDH, Hb and PSA were significantly related with poor survival; in addition, DE in bone also showed a negative relationship (Table 2).

## DISCUSSION

Adverse skeletal events often lead to a deterioration in the quality of life of prostate cancer patients (Weinfurt *et al*, 2005). In our series of prospectively followed bone metastatic patients with hormone-refractory disease, 43% experienced SREs, with 5% occurring before and 38% after the onset of hormone-refractory disease. The time to onset of the first adverse event in the hormone-refractory phase, although only 7 months on average, was distributed over a wide range (1–39 months). In all, 90% of patients died, 58.8% of which died of disease without experiencing an SRE. Among those still alive at the last follow-up appointment, five were still SREs-free more than 4 years since the date of hormone refractoriness.

The first comment to this study is that many patients are expected to develop SREs in the hormone-refractory phase of the disease, but they constitute a minority of those potentially at risk. In this respect, the hormone-refractory patients were a heterogeneous population, since SREs occurred within a few months in many patients or sometimes even years later in others.

Knowing whether the chance to develop SREs is high or low and whether an SRE may occur within months or years is useful for physicians when planning the frequency of follow-up investigations and guiding decisions about the choice of treatment. In addition, the identification of predictive factors for developing SREs may be a valuable aid in designing studies to test bone resorption inhibitors that can prevent adverse skeletal events, since an imbalance in these factors when comparing study arms could lead to biased results.

Zoledronic acid administration in hormone-refractory prostate cancer patients with metastatic bone disease has been demonstrated to significantly reduce the frequency of SREs. No patients in our series received zoledronic acid, but 31 had been treated with a single dose of pamidronate, another bisphosphonate (Berruti *et al*, 2002). Since pamidronate had failed to reduce SREs in the prostate cancer population (Lipton *et al*, 2002), these patients were not removed from the analysis.

A recent study pointed out that zoledronic acid administration in bone metastatic prostate cancer patients with hormone-refractory disease is not cost-effective since the costs of administering the drug outweigh the savings on the cost of medical care linked to the reduction in SREs (Reed *et al*, 2004). We believe, however, that the use of this drug might be more convenient if it were administered only to patients at risk of developing SREs.

Among the skeletal related parameters considered in the present study as predictive factors for SREs, disease estent in bone, bone pain, serum ALP and urinary NTx displayed a significant association in univariate analysis, while serum calcium and type of bone lesions did not. In a previous paper, we reported that urinary deoxypyridinoline, a bone resorption marker, showed a significant correlation with the probability to develop SREs (Berruti *et al*, 2000). In the present study, NTx also showed a similar relationship. *N*-telopeptide of type one collagen has recently been demonstrated to be an independent predictor of SREs in prostate cancer patients and those with non-small-cell lung cancer or other solid tumours (Brown *et al*, 2005). Our data are in line with these results. Unfortunately, this marker failed to be an independent predictor when adjusted for DE in bone. The relationship between NTx with DE in bone could provide a possible explanation for this failure. *N*-telopeptide of type one collagen, however, was available in about 50% of cases, so that caution should be taken when interpreting data coming from a subgroup analysis. Serum calcium is notoriously tightly regulated and so does not reflect the bone matrix break-down. This is probably the reason why it failed to show a predictive role in our series. The absence of a relationship between the type of bone lesions and the occurrence of SREs is somewhat surprising. It should be noted, however, that the small number of patients with lytic/mixed bone lesions may have limited the power of the analysis.

The multivariate analysis identified bone pain and DE in bone as two independent parameters predictive for the occurrence of SREs. By combining these two variables, dichotomised at median value, we defined three patient subgroups. The group with low pain score and limited DE in bone had a low incidence of SREs, whereas the group with high pain score and extensive tumour load in bone had a high incidence of SREs. The incidence of SREs in the patients group with either of these two predictors was intermediate. The time-to-first-SRE curves show that patients with heavy tumour load in bone and high pain score developed SREs much earlier than those with less tumour load and bone pain. This observation comes somewhat expectedly but has never been reported before. Disease extent in bone was found to be predictive for spinal cord compression in a small study on 68 cases, 61 of which had hormone-refractory disease (Bayley *et al*, 2001), thus confirming our observations. In the same study, however, the authors reported a direct relationship between the occurrence of spinal cord compression and the duration of androgen deprivation therapy, a finding that contrasts with our results.

Recognised prognostic factors such as Gleason score at diagnosis, Hb, LDH, PS and albumin (Smaletz *et al*, 2002; Halabi *et al*, 2003) were confirmed as being significantly related with poor survival but failed to be predictive for SREs; in addition, some factors predictive for SREs were also negative prognostic parameters. By combining prognostic parameters (Smaletz *et al*, 2002; Halabi *et al*, 2003) with predictive factors of SREs, it may be possible to identify a patient subset with a higher likelihood of dying before the onset of SREs in which bisphosphonate therapy could be potentially avoided.

In our series, the duration of androgen deprivation therapy significantly correlated with the occurrence of SREs in the hormone-sensitive phase of the disease, thus confirming its causative role (Daniell *et al*, 2000; Oefelein *et al*, 2002), but failed to show a predictive role when the disease become hormone refractory. In this study, benign SREs cannot be distinguished from malignant SREs, which may be an obstacle to assessing the real impact of iatrogenic SREs. This issues needs to be addressed in future studies. Noteworthy, however, is that the majority of SREs were distributed among patients with heavy tumour load in bone. These data suggest that in the hormone-refractory phase of the disease bone loss due to tumour-induced osteolysis prevail over an iatrogenic effect.

In a large series of men with prostate carcinoma prospectively followed after initiation androgen deprivation therapy until death, the duration of hormone therapy conferred an independent risk for SREs (Krupski *et al*, 2004). In this study, however, only skeletal fractures were considered as SREs, and no distinction was made between SREs occurring before or after the onset of hormone refractory disease.

In conclusion, prostate cancer patients with hormone-refractory disease are generally more likely to die of the disease than to develop SREs. Our study shows that bone pain intensity and DE in bone, two variables easily available in clinical routine, independently predict the occurrence of SREs in this patient population. By combining these parameters we found patient subsets with a different risk of SREs. These findings have several implications: (1) they could help physicians in planning the most appropriate skeletal follow-up for individual patients; (2) they suggest future trials aimed at delaying zoledronic acid administration in patients with low risk of developing SREs and/or avoiding drug delivery in patients with a high probability of dying of disease before the occurrence of SREs (ie patients with very limited disease in bone and concomitant visceral metastases). Finally, our results may prove useful for stratifying patients enrolled in clinical trials for testing the efficacy of new bone resorption inhibitors to prevent SREs.

## Figures and Tables

**Table 1:** Patient characteristics

No	200
Age median (year) (range)	73 (52–92)
	
*ECOG PS*	
0	42 (21.0%
1	91 (45.5%)
2–3	67 (33.5%)
	
*Gleason score at diagnosis (196 patients)*	
<8	95 (48.5%)
⩾8	101 (51.5%)
	
*Previous endocrine therapies*	
LHRH-A	182 (91.0%)
LHRH-A+antiandrogens	18 (9.0%)
	
PSA (ng ml^−1^) (198 patients)^a^	118.0 (0.1–9000.0)
Alkaline phosphatase (U l^−1^) (186 patients)^a^	165.0 (47.0–6000.0)
NTx (nmol mmol^−1^ creatinine) (103 patients)^a^	96.0 (11.6–662.0)
Ca (mmol l^−1^) (184 patients)^a^	2.35 (1.91–4.20)
Hb (g dl^−1^) (182 patients)^a^	12.1 (7.3–15.6)
Albumin (g dl^−1^) (170 patients)^a^	3.6 (1.9–6.7)
LDH (U l^−1^) (178 patients)^a^	405 (136–3011)
Androgen deprivation duration (months) (188 patients)^a^	24.5 (2.03–228.0)
Disease extent in bone (number of sites) (198 patients)^a^	6 (1–9 or more)
Pain score (200 patients)^a^	5 (0–16)
	
*Types of bone lesions (190 patients)*	
Lytic	14 (7.4%)
Mixed	42 (22.1%)
Blastic	134 (70.5%)

a Data are median (range).

**Table 2:** Proportion of adverse skeletal related events (SREs), relative risks (RRs) and 95% confidence intervals (CIs) associated with SREs or death

			**Time to first SRE**			**Time to death**		
	**Proportion SREs**	***P*-value**	**RRs**	**95% CI**	***P*-value**	**RRs**	**95% CI**	***P*-value**
*Performance status*								
0–1	47/135 (34.8%)	0.18	2.07	1.3–3.28	0.002	1.65	1.20–2.70	0.002
>1	29/65 (44.6%)							
								
*Gleason score*								
<8	38/95 (40.0%)	0.59	1.03	0.60–1.80	0.90	1.31	1.07–1.62	0.01
⩾8	38/101 (37.6%)							
								
*PSA (ng ml* ^−*1*^ *)*								
⩽118	37/100 (37.0%)	0.79	1.37	0.87–2.15	0.18	1.42	1.05–1.93	0.02
>118	38/98 (38.7%)							
								
*Hb (g dl* ^−*1*^ *)*								
⩽12.5	38/89 (42.7%)	0.66	0.89	0.56–1.42	0.63	0.61	0.43–0.88	0.003
>12.5	38/93 (40.8%)							
								
*Albumin (g dl* ^−*1*^ *)*								
⩽3.6	34/90 (37.7%)	0.66	0.85	0.53–1.36	0.49	0.64	0.46–0.88	0.007
>3.6	34/83 (40.9%)							
								
*LDH (U l* ^−*1*^ *)*								
⩽405	32/75 (42.6%)	0.62	1.21	0.73–2.01	0.46	1.61	1.13–2.28	0.008
>405	29/75 (38.6%)							
								
*Types of bone lesions*								
Lytic/mixed	25/56 (44.6%)	0.28	0.85	0.53–1.36	0.50	1.00	0.72–1.41	0.97
Blastic	51/134 (38.1%)							
								
*Disease extent in bone*								
⩽6 sites	28/94 (29.8%)	0.015	2.41	1.50–3.88	0.0001	1.47	1.08–2.01	0.014
>6 sites	48/103 (46.6%)							
								
*Bone pain*								
Pain score <5	31/105 (29.5%)	0.011	1.94	1.44–2.63	0.0001	1.53	1.27–1.85	0.0001
Pain score ⩾5	44/95 (45.8%)							
								
*ALP (U l* ^−*1*^ *)*								
⩽165	29/94 (30.8%)	0.014	2.50	1.56–4.02	0.0001	1.71	1.25–2.35	0.001
>165	45/93 (48.4%)							
								
*NTx (nmol mmol*^−*1*^ *creatinine)*								
⩽96	10/50 (20.0%)	0.001	3.10	1.47–6.54	0.001	1.37	0.90–2.06	0.14
>96	31/53 (58.5%)							
								
*Ca (mmol l* ^−*1*^ *)*								
⩽2.35	38/92 (41.3%)	0.65	0.64	0.35–1.17	0.15	1.10	0.80–1.51	0.54
>2.35	35/92 (38.0%)							
								
*Duration of androgen deprivation therapy (months)*								
⩽24.4	41/94 (43.6%)	0.37	0.65	0.41–1.02	0.06	0.74	0.54–1.01	0.05
>24.4	35/94 (37.2%)							

**Table 3:** Independent factors predictive for adverse skeletal events according to a multivariate Cox model

**Variables**	**Hazard ratio**	**95% confidence intervals**	***P*-value**
*Variables not in the model*			
Alkaline phosphatase	1.11	0.84–1.48	0.45
Performance status	1.22	0.70–2.12	0.47
			
*Variables in the model*			
Number of metastatic sites	1.16	1.07–1.25	0.000
Pain score	1.13	1.06–1.20	0.000
